# The effect of a training program on the self‐care efficacy of hemodialysis patients with mineral and bone disorders: A quasi‐experimental study

**DOI:** 10.1002/hsr2.1429

**Published:** 2023-07-12

**Authors:** Sedigheh Tashakor, Behnaz Bagherian, Zahra Salmanpour, Roghayeh Mehdipour‐Rabori

**Affiliations:** ^1^ Nursing Research Center, Department of Medical‐Surgical Nursing Kerman University of Medical Science Kerman Iran; ^2^ Nursing Research Center, Department of Medical‐Surgical Nursing, Razi Faculty of Nursing and Midwifery Kerman University of Medical Science Kerman Iran; ^3^ Department of Internal Medicine Fasa University of Medical Science Fasa Iran

**Keywords:** chronic kidney disease‐mineral and bone disorder, education, hemodialysis, self‐care

## Abstract

**Background and Aims:**

Patients who have chronic kidney disease (CKD) and mineral and bone disorders (MBD) often do not know much about their diseases. A training program can help them improve their quality of life. This study aimed to assess the effect of a training program on the self‐care efficacy of the hemodialysis patients with MBDs in southeastern Iran.

**Method:**

We conducted a quasi‐experimental study involving 49 patients with CKD‐MBD in southeastern Iran in 2021. The patients were randomly assigned to either the control or intervention group. The intervention group received 12 self‐care training sessions delivered through WhatsApp, whereas the control group received routine information. We administered CKD‐MBD knowledge and behavior questionnaires of the intervention, and measured laboratory parameters before and 1 month after the intervention. The data were analyzed by SPSS15 with descriptive and analytical statistics. Paired *t* test, independent *t*, analysis of covariance, and Mann–Whitney *U* tests were using for data analysis.

**Results:**

The mean knowledge scores of the control group were 4.78 ± 1.78 and 6.22 ± 2.11 before and after the intervention, respectively (*p* = 0.200), whereas the mean knowledge scores of the intervention group were 6.08 ± 2.24 and 22.23 ± 4.55 before and after the intervention, respectively (*p* = 0.001). The mean behavior scores of the control and intervention groups were 75.61 ± 7.13 and 73.85 ± 7.49 before the intervention, respectively (*p* = 0.070), but they received the mean scores of 78.87 ± 5.58 and 82.50 ± 5.35 after the intervention, respectively (*p* = 0.001). The result showed a significant increase in the mean knowledge and behavior scores after the intervention. The researchers found no significant difference in the mean scores of the laboratory parameters between them before and after the intervention (*p* = 0.090); therefore, the intervention could not affect the laboratory parameters.

**Conclusion:**

To sum up, the study found that the training program improved the knowledge and behavior of hemodialysis patients with MBD. WhatsApp was a good and cheap way to teach them self‐care, and it helped them do it better. These results implied that this training program could help the patients have a better quality of life.

## 1 INTRODUCTION

Chronic kidney disease (CKD) is a noncommunicable disease that continues to increase in prevalence.^1^ In the United States, ~37 million adults suffer from this disease.^2^ Globally, CKD affects 13.4% of the population,^2^, ^3^, ^4^, ^5^ with the highest prevalence found in Asia, accounting for 60% of cases worldwide. Studies have shown that 5%–15% or even up to 23% of the Iranian population may be affected by this disease.^6^

CKD is often accompanied by a mineral and bone disorder (MBD) referred to as CKD‐MBD. This disorder can affect the bones, heart, and blood vessels of individuals with CKD,^7^, ^8^ with 33%–67% of the patients with end‐stage CKD suffering from MBD; its prevalence increases as kidney damage progresses, leading to higher mortality rates.^9^ CKD‐MBD occurs when impaired kidney function leads to abnormal levels of calcium, phosphorous, parathyroid hormone, and Vitamin D in blood due to reduced mineral elimination. The secondary complications of renal failure include some abnormalities in bone turnover, mineralization, bone density, linear growth, and ectopic calcium in blood vessels and soft tissue.^10^

The kidneys play a crucial role in regulating phosphorous homeostasis, but abnormal levels of phosphorous can result in some complications in patients with CKD such as vascular calcification, bone and mineral disease, and hyperthyroidism.^11^ In addition, hyperparathyroidism secondary to chronic renal failure is a condition characterized by abnormal metabolism of calcium and phosphate and inappropriate secretion of parathyroid hormone. Abnormal mineral metabolism can lead to osteoporosis and vascular calcification, which can increase the risk of sudden cardiovascular problems and worsen the prognosis of the disease.^12^

Patients with CKD experience many complications that can significantly affect their daily lives;^13^ therefore, some measures are necessary to improve their quality of life, including increasing their knowledge and self‐care capabilities.^14^ The CKD knowledge is crucial for self‐management as recent evidence has shown that patients with CKD have insufficient knowledge of their disease and its treatment,^15^ so training plays a critical role in improving their knowledge. It is essential to recognize how actions can affect one's health behavior, particularly if it has turned into a habit.^16^

Self‐care training programs improve quality of life of the patients and their families and increase their participation in self‐care programs.^17^ These programs help patients to improve their self‐care abilities and personal commitment to their own health, leading to greater awareness and sound decisions about therapies and life changes, which can increase their emotional and physical well‐being.^18^ With the advancements in technology, virtual education can now be accompanied by text, voice, pictures and films, making it more accessible to patients. Social media have proven to be useful for exchanging health materials between patients and health professionals.^19^, ^20^ Therefore, this study aimed to investigate the impact of virtual training on the self‐care of CKD patients with MBDs, who were undergoing hemodialysis in southern Iran in 2021.

## 2 METHODS

### 2.1 Sample and setting

The present quasi‐experimental study was done in hemodialysis wards of Valiasr and Shariati hospitals in Fasa, Fars Province, Iran, in 2021; these two hospitals have 18 active beds and 106 patients undergoing hemodialysis for 8 or 12 h per week.

The inclusion criteria were the patients aged 18–67 years, who had been undergoing hemodialysis for at least 3 months and received hemodialysis 2 or 3 times per week. They had disrupted levels of calcium, phosphorous, or parathyroid hormone according to the physician's opinion, could read and write in the Persian language, had auditory and verbal abilities to answer questions, and could use smartphones and other virtual media. The exclusion criteria were patients with any psychiatric disorders or other diseases such as primary hyperparathyroidism, hypoparathyroidism, hyperthyroidism, bone and mineral disorder, who could not complete more than 10% of the questionnaire.

**Figure 1:**
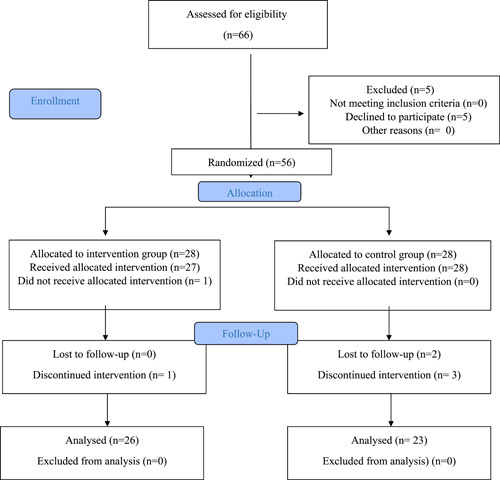
Standard flowchart for entry of participants.

A total of 106 patients were screened for the study, 66 of whom met the inclusion criteria, but 56 patients were willing to participate in the study. The patients were selected using the census sampling method and the consort flow diagram was attached (Figure 1). The patients were randomly assigned to either the intervention or control group by a nurse who was not aware of the patients and groups. One participant in the intervention group was excluded due to a malfunctioning smartphone and two participants in the control group were excluded, because they received kidney transplantation. In addition, one person in the intervention group and two in the control group passed away, whereas one person in the control group migrated, resulting in their exclusion from the study. Finally, 26 patients in the control group and 23 patients in the intervention group participated in the study.

The researcher observed that patients with CKD‐MBD had difficulty in concentrating and understanding the training provided by the personnel during and after hemodialysis. As these patients underwent hemodialysis three or four times a week, they could not assign extra time to participate in face‐to‐face training, so this training was conducted separately from the routine training during dialysis.

### 2.2 Intervention

The ward nurse provided routine information to the control group, whereas the intervention group received training on WhatsApp.

In the first session, the researcher explained the study goals, received consent forms, and asked participants to complete the questionnaires (demographic and CKD‐MBD knowledge and behavior [CKD‐MBD‐KB] questionnaire, and a checklist of laboratory parameters).

All participants in the intervention group were added to a WhatsApp group, where the researchers sent various educational materials, including PowerPoints, photos, videos, Microsoft word documents, and voices. The participants were trained on Sundays and Wednesdays over 12 sessions for 6 weeks. The researchers covered several topics, including renal failure and its etiology, hemodialysis mechanism and its benefits, the function of a hemodialysis machine, complications after hemodialysis and preventive strategies, fluid restriction, activity rate, provision of care for the vascular access site, diet, and MBD of kidney disease. For example, a video was prepared to train participants on proper nutrition.

One file was posted on the WhatsApp group during each session and the patients could ask their questions. The researchers checked and answered their questions in the mornings and evenings. Patients and their families had enough time to read the educational materials, which were very simple and easy‐to‐use for all age groups. The researchers sent regular reminders to the participants on WhatsApp or contacted them to open the educational files.

Nephrologists approved the content validity, whereas the researchers designed the educational content. The patients were allowed to ask their questions and interact with each other. A specialist replied to the questions if necessary.

The CKD‐MBD‐KB questionnaire was completed 1 month after the intervention. The patients’ clinical results were also sent and registered.

The researcher was present in the hemodialysis wards of Valieasr and Shariati hospitals to complete the questionnaires, because most of the patients had difficulty answering the questions due to the fistula fixed in their hands. The duration needed to complete the questionnaires was about 20 min.

### 2.3 Measures

The data collection tools included a demographic and CKD‐MBD‐KB questionnaire, and a checklist of laboratory parameters.

The demographic questionnaire included gender, marital status, age, position, education level, the duration of kidney disease, the duration of hemodialysis treatment, medications, and other diseases such as high blood pressure or diabetes.

We used CKD‐MBD‐KB questionnaire introduced by Shi et al.^21^ and consisted of three parts. The first part contained 23 items assessing disease‐related knowledge, diet‐related knowledge, and medicine‐related knowledge. Each item had two scores, with wrong and right answers receiving 0 and 2 scores, respectively. The total score of this part ranged from 0 to 46.

The second part consisted of 24 items assessing compliance and attitude, rated on a Likert scale ranging from one (never) to five (always). The total score of this part ranged from 0 to 120. The last section had three open‐ended questions to clarify the reasons for patients’ poor medication adherence, their points of view on the most qualified instructors for treatment, and the main factors for patient training.

Therefore, the CKD‐MBD‐KB questionnaire included 50 items with a single total score (166 points), with the higher scores representing patients’ higher knowledge, compliance, and attitude towards their disease.^21^

As the questionnaire had not been translated into Persian before this study, the research team measured the validity and reliability of this questionnaire. The respondents provided their consent via email, and the questionnaire was translated based on the protocol of the World Health Organization. The clarity of the translated questionnaire was tested by face‐to‐face interviews with five hemodialysis patients.

The patients had to express each item of the questionnaire with their own words and indicate how they chose their responses; this process was performed for all items of the questionnaire. As the sample size in the present study was small, it was considered as the study population. Finally, the Persian version of the questionnaire was provided to investigate its content validity. A faculty member fluent in the English language translated the questionnaire and 10 faculty members of the Razi School of Nursing revised it. The Cronbach's *α* coefficient was 0.864. The final version of the CKD‐MBD‐KB questionnaire had 3 domains, 5 facets, and 50 items. The reliability analysis showed that the Cronbach's *α* of the five facets ranged from 0.778 to 0.854, with retest correlation coefficients of the five facets ranging from 0.825 to 0.944.

In addition to the questionnaire, laboratory parameters were used to measure self‐efficacy.

The laboratory testing center used in this study was one of the well‐equipped centers in Fasa. A sample of tests was checked in this center and in another center, and the results were found to be the same. The person in charge of the hemodialysis department interpreted the tests. The laboratory parameters were measured for both groups using the same machine and method before and after the intervention. These parameters included the levels of calcium, phosphorous, parathyroid hormone, alkaline phosphatase, and 25‐hydroxyvitamin D. The study patients’ laboratory parameters received a score of 1 or 0, depending on whether they were normal or abnormal. If the result of the test was normal, a score of 1 was given, and if it was not normal, a score of 0 was given. Moreover, if all test results were either normal or abnormal, they received a score of 5 or 0, respectively. The total score was between 0 and 5, with a higher score indicating that most of the tests were normal.

### 2.4 Ethics approval and consent to participate

The Ethics Committee of the Kerman University of Medical Science approved this study with project No. 99000561 and code of ethics No IR.KMU.REC. 1399.477. The following ethical criteria were regarded: written consent form, study goal description, and confidentiality of data.

### 2.5 Statistical analysis

The data were analyzed by SPSS15 as well as descriptive and analytical statistics (frequency, mean, and SD). The significance level was considered 0.05. According to the Kolmogorov–Smirnov test, the distributions of the data was normal, so independent *t* test was used. The within‐group comparison was done using the paired *t* test, whereas the between‐group comparison was done by independent *t* test.

## 3 RESULTS

We divided 49 patients into the control and intervention groups. The mean ages of the participants in the control and intervention groups were 54.00 ± 9.52 and 52.54 ± 12.73, respectively. Most of the participants in the two groups were married. Table 1 showed no significant difference in demographic data between the two groups, so they were homogeneous in demographic data (Table 1).

**Table 1:** Comparison of demographic variables in the study groups.

Demographic information		Intervention		Control		
		Count (*n* = 26)	*N* %	Count (*n* = 23)	*N* %	*p*
Sex	Female	10	38.5%	13	56.5%	0.060^a^
	Male	16	61.5%	10	43.5%	
Marital status	Single	4	15.4%	1	4.3%	0.100^a^
	Married	22	84.6%	22	95.7%	
Economic status	Good	3	11.5%	1	4.3%	0.070^b^
	Moderate	8	30.8%	8	34.8%	
	Poor	15	57.7%	14	60.9%	
Education	Middle/high school	13	50.0%	11	47.83%	0.080^a^
	Diploma/higher	13	50.0%	12	52.17%	
Job	Employed	10	38.5%	10	43.5%	0.100^a^
	Unemployed	16	61.5%	13	56.5%	

^a^ The *χ* ^2^ test.

^b^ Fisher exact test.

The mean knowledge scores of the control group were 4.78 ± 1.78 and 6.22 ± 2.11 before and after the intervention, respectively (*p* = 0.200), whereas the mean knowledge scores of the intervention group were 6.08 ± 2.24 and 22.23 ± 4.55 before and after intervention, respectively (*p* = 0.001). The mean difference score of knowledge in the intervention group was 16.15. The results indicated that the training program improved the main knowledge scores in the intervention group (Table 2).

**Table 2:** Comparison of CKD‐MBD knowledge in the study groups before and 1 month after the intervention.

Knowledge	Intervention		Control		
	Mean	SD	Mean	SD	*p*
Before intervention	6.08	2.24	4.78	1.78	0.060^a^
After intervention	22.23	4.55	6.22	2.11	0.001^a^
Difference before and after the intervention	16.15	2.31	1.44	0.33	0.001^a^
*p*	0.001^b^		0.200^b^		

Abbreviation: CKD‐MBD, chronic kidney disease associated with mineral and bone disorder.

^a^ *T* test.

^b^ Paired *T* test.

Our results indicated that the mean behavior scores of the control and intervention groups were 75.61 ± 7.13 and 73.85 ± 7.49 before the intervention, respectively (*p* = 0.070), but the mean behavior scores of the intervention group were 73.85 ± 7.49 and 82.50 ± 5.35 before and after the intervention, respectively (*p* = 0.001). The results indicated that the training program improved the main behavior scores in the intervention group (Table 3).

**Table 3:** Comparison of CKD‐MBD behavior in the study groups before and 1 month after the intervention.

Behavior	Intervention		Control		
	Mean	SD	Mean	SD	*p*
Before intervention	73.85	7.49	75.61	7.13	0. 070^a^
After intervention	82.50	5.35	78.87	5.58	0.002^a^
Difference before and after the intervention	8.65	7.05	3.26	4.09	0.001^a^
*p*	0.001^b^		0.070^b^		

Abbreviation: CKD‐MBD, chronic kidney disease associated with mineral and bone disorder.

^a^ *T* test.

^b^ Paired *T* test.

The mean total scores of the knowledge and behavior in the control group were 80.39 ± 8.91 and 85.09 ± 7.69 before and after the intervention, respectively, whereas the mean total scores in the intervention group were 79.93 ± 9.73 and 104.73 ± 11.09 before and after intervention, respectively (*p* = 0.001).

The results indicated that the mean scores of laboratory parameters in the control and intervention groups were 2.26 ± 0.81 and 2.23 ± 0.71 before the intervention, respectively; we found no significant difference in the mean scores of the laboratory parameters between them before and after the intervention (*p* = 0.090) (Table 4). The results of the study indicated no significant change in the laboratory parameters between the two groups before and after the intervention. As the *p*‐value was found to be 0.090, it indicates that the observed difference may have arisen by chance. Therefore, we cannot conclude that the intervention had a significant impact on the laboratory parameters.

**Table 4:** Comparison of laboratory parameters in the study groups before and 1 month after the intervention.

Laboratory parameters	Intervention		Control		
	Mean	SD	Mean	SD	*p*
Before intervention	2.23	0.71	2.26	0.81	0.100^a^
After intervention	2.88	0.91	2.91	0.79	0.090^a^
Difference before and after the intervention	0.65	0.89	0.65	0.88	0.100^a^
*p*	0.100^b^		0.090^b^		

^a^ *T* test.

^b^ Paired *T* test.

## 4 DISCUSSION

The study aimed to assess the effect of a training program on the self‐care efficacy of hemodialysis patients with MBDs in southeastern Iran. The present study indicated the positive impact of training through social media on the knowledge and behavior of the patients undergoing hemodialysis. Hosseini et al.^22^ showed that nursing consultation through social media improved the self‐efficacy and weight control of patients undergoing hemodialysis. Their study highlights the importance of self‐efficacy in reducing complications, hospital stay, and treatment costs associated with hemodialysis.^22^ Evidence suggests that education can empower patients by improving their knowledge and skills, leading to better disease management and improved health outcomes in chronic diseases.^17^, ^18^

Alanzi et al.^23^ found that WhatsApp was effective in improving the self‐efficacy and knowledge of patients with diabetes, whereas Ahmed et al.^24^ reported that a structured training program improved the knowledge and self‐management behaviors of hemodialysis patients.

The study results showed no significant difference in the mean behavior scores between the two groups before the intervention but indicated a significant difference between them after intervention, supporting the impact of virtual training on the behavior in hemodialysis patients with CKD‐MBD.

Ramazani et al.^25^ indicated that a self‐efficacy theory‐based training program improved self‐care behaviors in hemodialysis patients and Lee et al.^26^ concluded that a self‐management training program could improve self‐care behaviors related to quality of life in patients with CKD.

Choi et al.^27^ studied the effect of a face‐to face self‐management program on the knowledge, self‐care behaviors, and kidney function of patients with CKD before renal replacement therapy.

The study results found that the mean scores of laboratory parameters in the control and intervention groups were not significantly different before and after the intervention.

Sandlin et al.^28^ revealed that nurse‐led education could increase patients’ phosphate binder adherence, but did not necessarily improve serum phosphate levels. This is consistent with the present study regarding the ineffectiveness of education on levels of calcium, phosphorous, and parathyroid hormone in study participants.

Chan et al.^29^ in Malaysia demonstrated that multidisciplinary education was an effective way in managing hyperphosphatemia in hemodialysis patients. A physician, pharmacist, and nutritionist conducted the intervention, which included group seminars and individual consultations over a 3‐month period and resulted in improved adherence to medication, knowledge, and levels of calcium, phosphorous, hemoglobin and ALP.^29^ It should be noted that the study design and educational method differed from our study.

Stumm et al.^30^ investigated the effect of nursing education on reducing hyperphosphatemia among patients undergoing hemodialysis. They showed a significant difference in the mean phosphorous score of study patients at the start of the intervention and 30 days after intervention. However, the comparison of the results showed no reduction in the mean score of phosphorus levels 30 and 60 days after intervention. They also indicated a significant increase in calcium levels of study samples before intervention, and 30 and 60 days after intervention, as well as no significant difference in alkaline phosphatase after intervention. However, the mean scores of parathyroid hormone (PTH) were different between the groups. Their study revealed that a nursing education program was effective in decreasing phosphate levels and itching of hyperphosphatemia patients,^30^ but it was inconsistent regarding the impact of education on phosphorous, PTH and calcium levels. This difference could be due to the type of interventions and the interval of measuring laboratory parameters. The present study revealed that virtual education could increase patients’ self‐care. Although previous studies have mostly focused on hemodialysis patients, limited studies are available on MBDs, which is an important issue for dialysis patients. The above studies confirmed that education could increase knowledge and change the behaviors of dialysis patients with MBDs. With the world experiencing a rise in online learning during the coronavirus disease 2019 (COVID‐19), our study adds to the growing body of evidence supporting the effectiveness of virtual education in improving patient self‐care.

## 5 LIMITATION

The present study had two limitations that should be acknowledged. First, the mental and emotional conditions of participants due to illness could have affected their responses, despite the researchers’ efforts to ask them to complete the questionnaires when they were mentally and emotionally ready. Second, the sample size in the present study was small, so the researchers recommend further studies with larger sample sizes.

## 6 CONCLUSION

This study demonstrated the positive impact of virtual education on the self‐care of hemodialysis patients with CKD‐MBD. Although we found no significant difference in laboratory parameters between the two groups before and 1 month after intervention, we had to measure laboratory parameters in longer intervals after the intervention. This study suggests that social media platforms can be effective tools in educational programs due to their simplicity, accessibility, affordability, and cost‐effectiveness, particularly among rural patients. This finding has important implications for healthcare providers, especially during the COVID‐19 pandemic, as virtual education can increase the knowledge of patients receiving hemodialysis.

**What is already known about the topic?**

- Most hemodialysis patients with mineral and bone disorders had low self‐efficacy.
- Patients could improve their self‐efficacy through education.
- Face‐to‐face education was unavailable because of the lack of staff and busy nurses, so other educational methods could be more effective.

**What this study adds?**

• Use of self‐care education through social media is an effective method for improving the level of knowledge and behavior of the hemodialysis patients with mineral and bone disorders, and it is more effective than the traditional method.

• As most patients have smartphones, nurses can train their patients through mobile phones.

## AUTHOR CONTRIBUTIONS

**Sedigheh Tashakor**: Conceptualization; data curation; funding acquisition; methodology; project administration; software; validation; visualization; writing—original draft; writing—review and editing. **Behnaz Bagherian**: Conceptualization; formal analysis; funding acquisition; investigation; resources; visualization; writing—original draft. **Zahra Salmanpour**: Conceptualization; formal analysis; funding acquisition; investigation; resources; visualization; writing—original draft. **Roghayeh Mehdipour‐Rabori**: Conceptualization; data curation; funding acquisition; investigation; methodology; project administration; software; supervision; validation; visualization; writing—original draft; writing—review and editing.

## CONFLICT OF INTEREST STATEMENT

The authors declare no conflict of interest.

## TRANSPARENCY STATEMENT

The lead author Roghayeh Mehdipour‐Rabori affirms that this manuscript is an honest, accurate, and transparent account of the study being reported; that no important aspects of the study have been omitted; and that any discrepancies from the study as planned (and, if relevant, registered) have been explained.

## Data Availability

The data sets used and/or analyzed during the current study are available from the corresponding author on reasonable request.
